# High diagnostic value of second generation CSF RT-QuIC across the wide spectrum of CJD prions

**DOI:** 10.1038/s41598-017-10922-w

**Published:** 2017-09-06

**Authors:** Alessia Franceschini, Simone Baiardi, Andrew G. Hughson, Neil McKenzie, Fabio Moda, Marcello Rossi, Sabina Capellari, Alison Green, Giorgio Giaccone, Byron Caughey, Piero Parchi

**Affiliations:** 1 Department of Biomedical and Neuromotor Sciences, University of Bologna, Bologna, Italy; 2 LPVD, Rocky Mountain Laboratories, NIAID, NIH, Hamilton, MT USA; 3 National CJD Research and Surveillance Unit, University of Edinburgh, Edinburgh, Scotland UK; 4 IRCCS Foundation Carlo Besta Neurological Institute, Milan, Italy; 5 IRCCS, Institute of Neurological Sciences, Bologna, Italy

## Abstract

An early and accurate *in vivo* diagnosis of rapidly progressive dementia remains challenging, despite its critical importance for the outcome of treatable forms, and the formulation of prognosis. Real-Time Quaking-Induced Conversion (RT-QuIC) is an *in vitro* assay that, for the first time, specifically discriminates patients with prion disease. Here, using cerebrospinal fluid (CSF) samples from 239 patients with definite or probable prion disease and 100 patients with a definite alternative diagnosis, we compared the performance of the first (PQ-CSF) and second generation (IQ-CSF) RT-QuIC assays, and investigated the diagnostic value of IQ-CSF across the broad spectrum of human prions. Our results confirm the high sensitivity of IQ-CSF for detecting human prions with a sub-optimal sensitivity for the sporadic CJD subtypes MM2C and MM2T, and a low sensitivity limited to variant CJD, Gerstmann-Sträussler-Scheinker syndrome and fatal familial insomnia. While we found no difference in specificity between PQ-CSF and IQ-CSF, the latter showed a significant improvement in sensitivity, allowing prion detection in about 80% of PQ-CSF negative CJD samples. Our results strongly support the implementation of IQ-CSF in clinical practice. By rapidly confirming or excluding CJD with high accuracy the assay is expected to improve the outcome for patients and their enrollment in therapeutic trials.

## Introduction

Human transmissible spongiform encephalopathies (TSEs) or prion diseases are neurodegenerative disorders characterized by the conversion of a constitutively expressed cellular glycoprotein, the prion protein (PrP^C^), into an abnormally folded, beta-sheet enriched, isoform (PrP^Sc^)^1^. While the mechanism of initial PrP^Sc^ formation remains largely unexplained, compelling evidence indicates that disease propagation involves the templated misfolding of PrP^C^ by PrP^Sc^ ^2, 3^.

Human prion diseases are highly heterogeneous disorders including four major disease groups, namely Creutzfeldt-Jakob disease (CJD), fatal insomnia, Gerstmann-Sträussler-Scheinker (GSS) syndrome, and variably protease-sensitive prionopathy (VPSPr)^4–7^. Disease subtypes with distinctive molecular and phenotypic features can also be found within these four groups, as it is exemplified by the current recognition of six clinico-pathological subtypes of sporadic CJD (sCJD) correlating at molecular level with the genotype at the polymorphic codon 129 (methionine, M or valine, V) in the gene encoding the prion protein (*PRNP*) and the type (1 or 2) of PrP^Sc^ accumulating in the brain^8, 9^. This phenotypic diversity mostly relates to the biology of prions, which exist in different strains, thought to be enciphered in distinct PrP^Sc^ conformations, that are able to transmit distinctive phenotypic traits, including incubation time, clinical signs, progression rate, type and patterns of PrP^Sc^ deposition, and neuropathological lesions^10, 11^. Specifically, current evidence indicate that five out of six sCJD subtypes (MM1, MM2C, MM2T, VV1 and VV2) behave as distinct prion strains after serial transmission into animal models. As the only exception, the VV2 and MV2K variants showed the same transmission properties, indicating a host-genotype (codon 129) effect^12–15^.

Due to of the significant phenotypic overlap with a number of other medical conditions which present with a rapidly progressive neurological syndrome, the clinical diagnosis of prion disease is often challenging. The introduction of diagnostic investigations such as brain diffusion weighted-MRI (DW-MRI) and surrogate CSF biomarkers such as the 14-3-3 and t-tau proteins has significantly increased the clinical diagnostic confidence^16, 17^. However, because of the lack of full specificity of these investigations, the post-mortem demonstration of PrP^Sc^ in the CNS tissue is still required for the definitive diagnosis of prion disease^18^.

Recently, the Real-Time Quaking-Induced Conversion (RT-QuIC) assay has been proposed as the first specific *in vivo* diagnostic test for sCJD^19^. By exploiting the self-replicating (seeding) power of pathogenic PrP^Sc^, RT-QuIC ultra-sensitively detects limited amounts of abnormal protein in CSF and other tissue samples^20^. The RT-QuIC technology builds on aspects of earlier QuIC^21, 22^ and amyloid seeding assays^23^, to provide high PrP^Sc^ sensitivity and specificity in a high throughput multiwell plate format with fluorescence detection. Results obtained in several laboratories with the first generation of this assay (PQ-CSF), mainly using full-length (23–231) hamster recombinant prion protein (rPrP^sen^) as the substrate, demonstrated a very high specificity but a suboptimal sensitivity, especially in sCJD subtypes associated with PrP^Sc^ type 2^24–27^. However, Orrù *et al*.^28^ recently introduced an improved, second generation RT-QuIC for CSF (IQ-CSF) assay which uses a truncated form of hamster recombinant PrP (rPrP^sen^, amino acids 90–231) as a substrate and other experimental conditions. Initial evaluation of the IQ-CSF assay indicated greater analytical and diagnostic sensitivity, and markedly shorter testing times^29–31^.

In this study, we contribute our experience with the second generation IQ-CSF in a large patient cohort including the largest patient population analysed to date affected by atypical and rare disease subtypes. Furthermore, to explore the performance of IQ-CSF in full, we provide a direct comparison between PQ- and IQ- methodologies, focusing on a largest group of definite CJD CSF samples analysed to date that were previously tested negative by PQ-CSF.

## Results

We investigated a total of 339 patients, including 239 CSFs from subjects with a definite (166) or probable (73) diagnosis of prion disease and 100 from patients with a definite alternative diagnosis (non-CJD). Specific aims of the study included: (i) the direct comparison of the overall performance of IQ-CSF with respect to the first generation RT-QuIC (PQ-CSF); (ii) the evaluation of the diagnostic accuracy of IQ-CSF across the spectrum of human prion disease subtypes; and (iii) the determination of the diagnostic performance of IQ-CSF in the clinical setting. Demographic data and investigative findings of the whole tested cohort are summarized in Table 1.

**Table 1:** Demographic features and results of diagnostic investigations in the tested patient cohort.

**Classification**	**n**	**Demographic data**			**CSF**		Brain MRI*	**EEG**
		**Gender** (n of F) [%]	**Mean age at onset** (years)	**Mean disease duration** (months)	**14-3-3 protein** Positive/Tested [%]	**t-tau > 1250 pg/mg** Positive/Tested [%]	Positive/Tested [%]	PSWCs/Tested [%]
**Definite sCJD**	116	63 [54.3]	65.9 ± 10.5	10.1 ± 11.6	89/112 [79.5]	92/107 [85.9]	74/89 [83.1]	39/107 [36.4]
MM1	43		67.5 ± 7.9	3.3 ± 2.2	41/43 [95.3]	40/42 [95.2]	29/35 [82.8]	29/40 [72.5]
VV2	33		58.2 ± 8.7	6.9 ± 2.4	31/31 [100.0]	29/29 [100.0]	21/24 [87.5]	4/30 [13.3]
MV2K	26		65.6 ± 10.2	20.3 ± 16.1	14/25 [56.0]	19/24 [79.2]	15/17 [88.2]	3/23 [13.0]
MM2C	9		60.1 ± 15.8	21.5 ± 14.4	2/8 [25.0]	2/7 [28.6]	7/8 [87.5]	3/9 [33.3]
MM2T	4		42.0 ± 14.5	27.0 ± 7.9	0/4 [0.0]	1/4 [25.0]	1/4 [25.0]	0/4 [0.0]
VV1	1		64.0	13.5	1/1 [100.0]	1/1 [100.0]	1/1 [100.0]	0/1 [0.0]
**Probable sCJD**	73	39 [53.4]	68.1 ± 8.0	8.9 ± 7.4	62/73 [84.9]	69/73 [94.5]	56/65 [86.1]	27/65 [41.5]
MM	39		67.2 ± 8.9	5.1 ± 5.2	37/39 [94.9]	38/39 [97.4]	31/36 [86.1]	23/35 [65.7]
MV	24		68.3 ± 7.4	14.2 ± 7.8	15/24 [62.5]	21/24 [87.5]	17/19 [89.5]	4/21 [19.0]
VV	10		71.1 ± 5.3	7.5 ± 4.2	10/10 [100.0]	10/10 [100.0]	8/10 [80.0]	0/9 [0.0]
**Genetic CJD** ^§^	33	22 [66.7]	60.6 ± 10.1	9.6 ± 11.5	27/33 [81.8]	31/33 [93.9]	18/23 [78.3]	9/23 [39.1]
**FFI**	2		50.0 ± 7.1	23, NA	0/2 [0.0]	0/1 [0.0]	0/1 [0.0]	0/1 [0.0]
**GSS**	6		44.0 ± 11.1	48 ± 12°	1/6 [16.7]	2/5 [40.0]	1/6 [16.7]	0/5 [0.0]
**Definite vCJD**	4		34.7 ± 10.4	17.5 ± 12.0	2/4 [50.0]	1/4 [25.0]	2/4 [50.0]	0/4 [0.0]
**Definite iCJD**	2		54.5 ± 31.8	20.0 ± 24.0	2/2 [100.0]	2/2 [100.0]	1/1 [100.0]	0/1 [0.0]
**Definite VPSPr**	3		71.3 ± 5.0	24.7 ± 10.1	2/3 [66.7]	1/1 [100.0]	0/3 [0.0]	0/2 [0.0]
**Non-CJD**	100	49 [49.0]	67.3 ± 13.2	17.9 ± 33.5	39/98 [39.8]	30/93 [32.2]	15/72 [20.8]	20/80 [25.0]

^*^According to Zerr *et al*.^18^, MRI findings were considered positive when showing (either in DW or FLAIR sequences) a hyperintensity in the striatum or in at least two cortical regions; therefore only MRI studies including DW and/or FLAIR sequences were taken into account. ^§^Genetic CJD cases included the following *PRNP* haplotypes: E200K-129M, V210I-129M, D178N-129V, R208H-129V, and E219G-129V. °3 patients are still alive.

### Comparison of IQ-CSF and PQ-CSF performance

To this aim we used 163 cases with a probable or definitive diagnosis of prion disease and 100 non-CJD cases. Samples were largely selected based on previous results by PQ-CSF. Most significantly, they included all prion CSF samples available to us (n = 93) that were either negative or only partially positive (2 out of 4 positive wells) by PQ-CSF (Suppl. Table 1).

Using IQ-CSF, 47 of the 58 cases that were negative by PQ-CSF showed a positive seeding activity (Suppl. Table 1). Among the 11 that remained negative under IQ-CSF conditions, 4 showed a significant blood contamination; the other 7 included 1 FFI, 1 GSS P102L, 1 sCJD MM1, 1 sCJD MV2K, 2 sCJD MM2C and 1 probable sCJD (MV).

The analysis of the 104 samples that were positive in both assays revealed a significant increase in the percentage of positive wells under IQ-CSF conditions (Suppl. Table 1).

**Figure 1:**
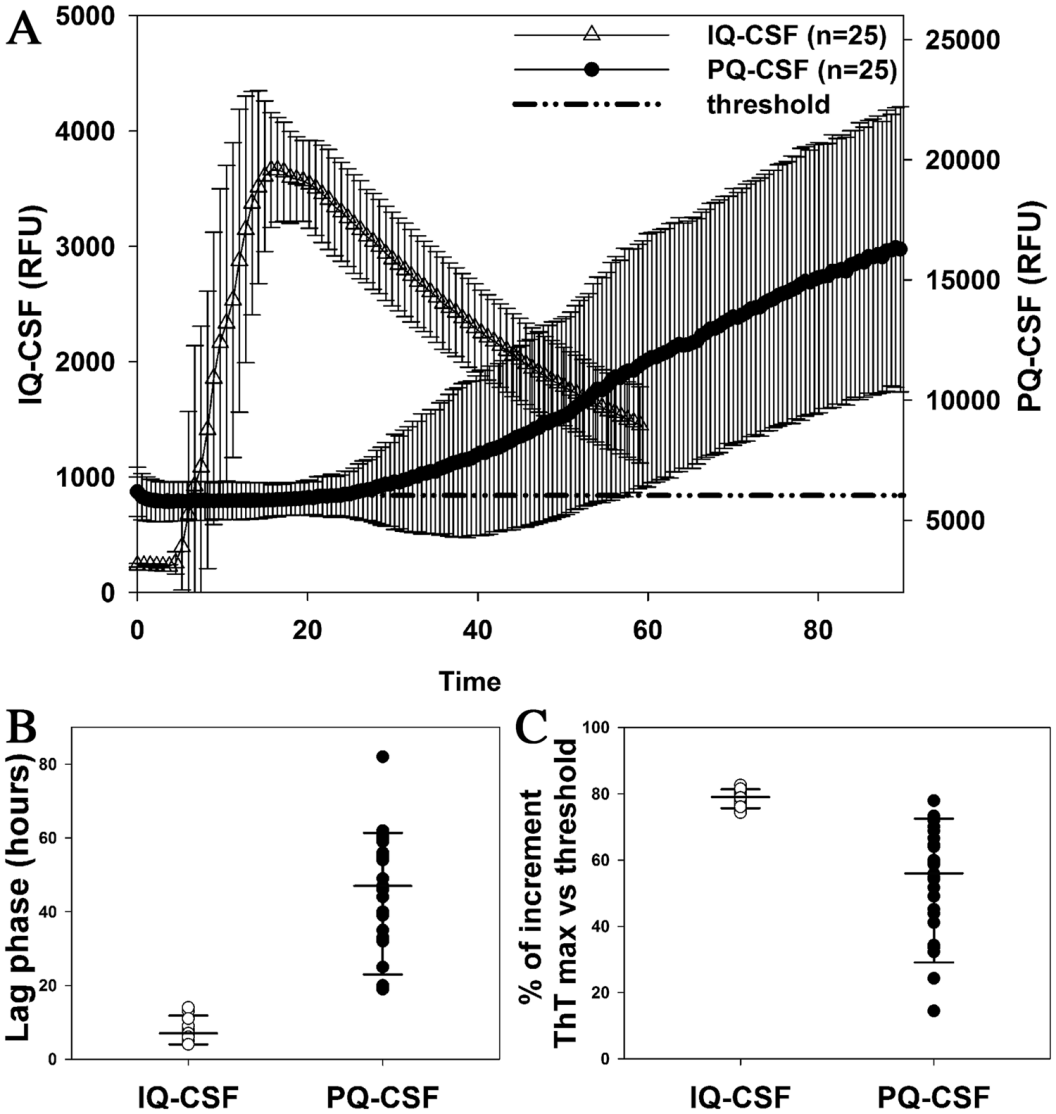
Comparison of kinetics and time to threshold between IQ-CSF and PQ-CSF RT-QuIC assays. (**A**) Averaged fluorescence kinetics of 25 sCJD (12 MM1, 8 VV2 and 5 MV2K) CSFs under, respectively, IQ-CSF (white) and PQ-CSF (black) conditions. Traces represent the mean ± SD of ThT fluorescence, the dot line the positivity threshold for both assays. (**B**) Comparison of the time to reach the threshold (lag phase) by IQ-CSF and PQ-CSF (n = 25 sCJD, p < 0.001). (**C**) Percentage of increment of ThT fluorescence with respect to the threshold by IQ-CSF and PQ-CSF (n = 25 sCJD, p < 0.001).

Furthermore, the comparison of the fluorescence curve responses in a group of 25 sCJD patients yielding a positive seeding activity under both assay conditions (Fig. 1A) confirmed that IQ-CSF is associated with a marked reduction of the mean lag phase (Fig. 1B, IQ-CSF = 7.6 ± 0.5 hours; PQ-CSF = 46 ± 3, n = 25, p < 0.001) and a significantly higher fluorescence response (Fig. 1C, IQ-CSF = 78.8 ± 0.4%; PQ-CSF = 53.0 ± 3.0%; n = 25, p < 0.001).

To determine whether IQ-CSF can detect a difference in seeding activity between prion samples that were either negative or positive under PQ-CSF conditions, we also compared the IQ-CSF fluorescence curves (Suppl. Figure 1A) between PQ-positive/IQ-positive and PQ-negative/IQ-positive samples. Interestingly, PQ-positive/IQ-positive samples had a lower lag phase (8.3 ± 0,3 vs 11.5 ± 0.9; n = 104, 47; p < 0.05; Suppl. Figure 1B), a higher ThT max (3816 ± 64 vs 3042 ± 106; n = 104, 47; p < 0.001; Suppl. Figure 1B), and a higher area under the curve (AUC) (124000 ± 2300 vs 102000 ± 4800; n = 104, 47; p < 0.001; Suppl. Figure 1B).

Finally, no sample from the non-CJD group (Table 2) yielded a positive response, resulting in a test specificity of 100%.

In summary, the present results confirm that IQ-CSF is associated with a significant increase in test speed and sensitivity while maintaining the very high specificity of PQ-CSF. Remarkably, IQ-CSF allowed the identification of 81% of those CJD samples which had previously tested negative by PQ-CSF.

### Analysis of IQ-CSF sensitivity across the spectrum of human prions

**Figure 2:**
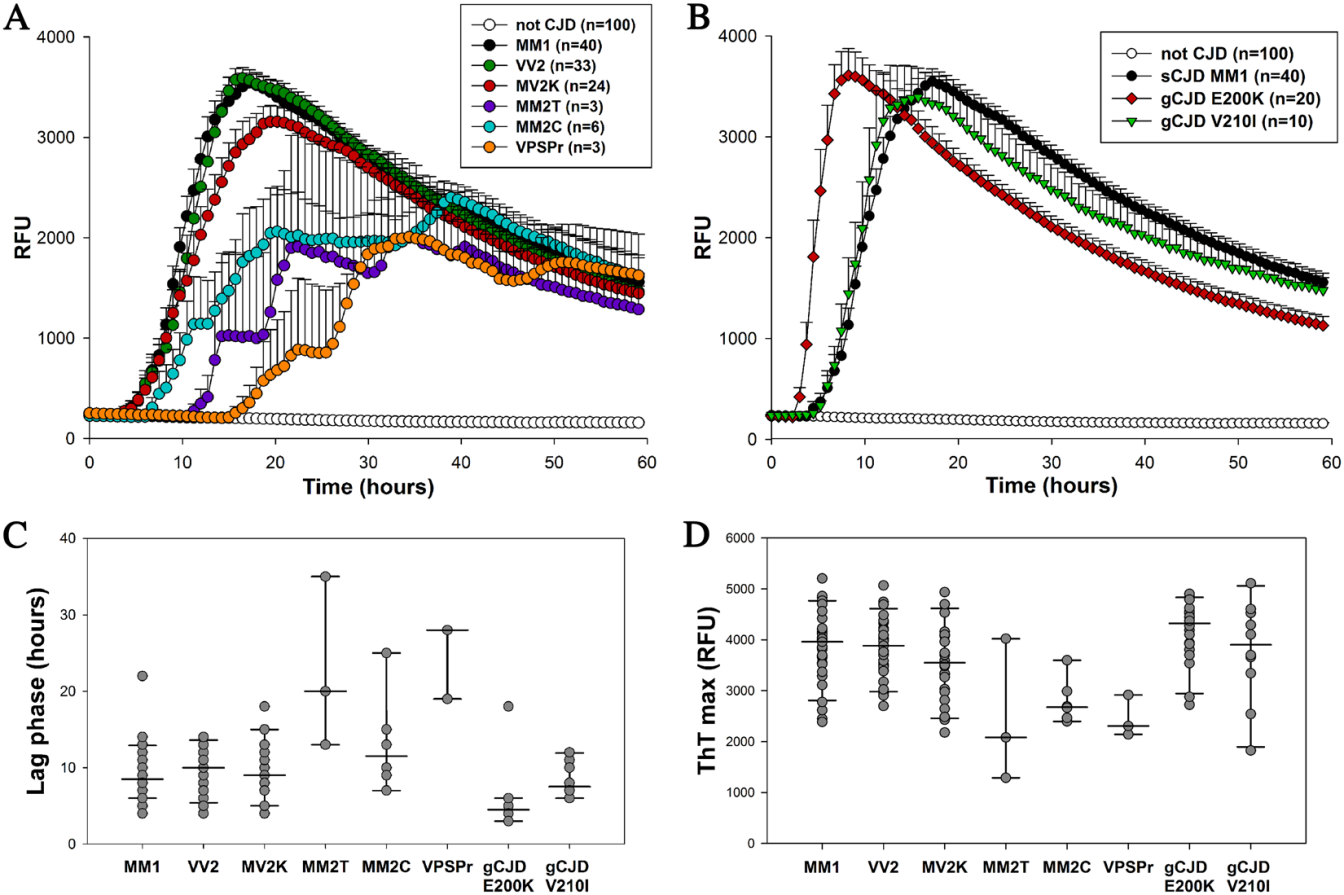
IQ-CSF RT-QuIC assay sensitivity in sporadic, genetic and acquired prion diseases according to CJD subtype or *PRNP* mutation. The upper boxes show the ThT fluorescence traces of (**A**) CJD subtypes MM1 (n = 40), VV2 (n = 33), MV2K (n = 24), MM2T (n = 3), MM2C (n = 6), VPSPr (n = 3), non-CJD (n = 100) and (**B**) CJD subtypes MM1 (n = 40), gCJD E200K-129M (n = 20), and gCJD V210I-129M (n = 10), non-CJD (n = 100). Data are expressed as mean ± SEM. The histograms and/or the scatter plot graphs show the lag phases (**C**) and ThT max (**D**) of the same disease groups.

To this aim we evaluated the IQ-CSF results in a panel of 239 samples, representative of all major human prion disease subtypes (Table 1). The results of the IQ-CSF assay for these different diagnostic groups are summarized in Table 3 and Fig. 2. Remarkably, the assay detected PrP^Sc^ with a very high sensitivity across the whole spectrum of sporadic CJD prions, with only minor variations among disease subtypes (Table 3 and Fig. 2A). Specifically, the test showed a 92 to 100% sensitivity for each of the three most common sCJD types (e.g. MM1, VV2, and MV2K), representing overall more than 90% of all CJD patients, and a lower, sub-optimal sensitivity only for the rare MM2C and MM2T sCJD subtypes. Interestingly, the IQ-CSF was also positive in all tested VPSPr cases.

**Table 2:** Diagnostic categories of non-CJD cases.

Diagnostic categories	14-3-3 protein positive/tested (%)	t-tau > 1250 pg/ml positive/tested (%)	brain MRI positive/tested (%)	Definite diagnosis (n)	
				pathological	clinical*
Alzheimer’s disease	4/17 (23.5)	4/15 (26.7)	2/13 (15.4)	16	1
Lewy body dementia	1/11 (9.1)	2/11 (18.2)	0/6 (0.0)	12	
Other neurodegenerative diseases°	2/6 (33.3)	3/6 (50.0)	1/6 (16.7)	4	2
Vascular dementia	6/10 (60.0)	4/9 (44.4)	2/7 (28.6)	10	
CNS malignancy	5/7 (71.4)	4/7 (57.1)	2/3 (66.7)	7	
Encephalitis (infectious or autoimmune)	12/28 (42.8)	9/28 (32.1)	5/23 (21.7)	10	18
Toxic/metabolic encephalopathies	4/9 (44.4)	4/10 (40.0)	1/7 (14.3)	6	4
No distinctive neuropathology (PrP^Sc^ negative)	4/10 (40.0)	0/7 (0.0)	2/7 (28.6)	10	

*Criteria for the definitive clinical diagnosis are provided in the methods.

°Other neurodegenerative diseases include Huntington disease (n = 1), corticobasal degeneration (n = 1), progressive supranuclear palsy (n = 1), frontotemporal dementia (n = 1), argyrophilic grain disease (n = 1), and primary age-related tauopathy (n = 1).

The comparison of the maximum fluorescence response (ThT max) among sporadic disease subtypes revealed a statistically significant difference between MM1 and MM2C (3900 ± 100, n = 40 vs 2800 ± 200, n = 6, p < 0.05) and between MM1 and VPSPr (3900 ± 100, n = 40 vs 2500 ± 230, n = 3, p < 0.05) (Fig. 2C). Similarly, the comparison of lag phases showed a statistically significant difference between MM1 and MM2C (8.9 ± 0.5, n = 40 vs 13.3 ± 3, n = 6, p < 0.05) and between MM1 and VPSPr (8.9 ± 0.5, n = 40 vs 25.0 ± 3, n = 3, p < 0.05) (Fig. 2D).

Regarding gCJD, IQ-CSF detected the prion seeding activity even in patients carrying rare *PRNP* haplotypes associated with atypical disease phenotypes, such as R208H-129V, E219G-129V, and D178N-129V (Suppl. Table 2). More significantly, between the two gCJD groups with at least 10 cases (Fig. 2B), the E200K-129M showed a higher ThT max mean value and a shorter lag phase in comparison to V210I-129M (ThT max = 4100 ± 130, n = 20 vs 3800 ± 300, n = 10; Lag phase = 5.2 ± 0.7, n = 20 vs 8.5 ± 0.7 n = 10, p < 0.05). Moreover, the lag phase was significantly shorter in E200K-129M in comparison to sCJD MM1, MV2K and VV2 (p < 0.05).

Finally, the results we obtained in a limited number of cases affected by rare genetic and acquired prion diseases such as GSS, FFI and vCJD suggest a rather low sensitivity of the assay for these variants.

In summary, we demonstrated the high sensitivity of IQ-CSF across the spectrum of human prions with a sub-optimal sensitivity for the MM2C and MM2T, and a low sensitivity limited to variant CJD, Gerstmann-Sträussler-Scheinker syndrome and fatal familial insomnia. Furthermore, sCJD MM2C and VPSPr prions appears to have a lower RT-QuIC seeding potency in comparison to the other most common sCJD subtypes (sCJD MM1, VV2 and MV2K). Finally, similarly to PQ-CSF, the IQ assay demonstrated that gCJD E200K-129M is the human prion disease subtype associated with the shortest lag phase.

### Overall performance of IQ-CSF in a “clinically” representative CJD population

To estimate the diagnostic performance of IQ-CSF in a clinical setting, we analyzed the assay results in a group of 187 unselected cases with a definitive neuropathologic or clinical diagnosis (either CJD or non-CJD), which were submitted to our Lab between January 2011 and March 2017. The results obtained for these groups are summarized in suppl. Table 3. At the chosen RFU threshold, 141 of the 145 CJD cases had a positive assay. The calculated test sensitivity was 97.2%, while the test’s positive predictive value (PPV) and negative predictive value (NPV) were 100% and 91.3% respectively.

**Table 3:** Sensitivity of IQ-CSF in sCJD, gCJD and other rare prion disease variants.

Disease variants	n	Positive	Negative	Sensitivity (%)
***definite sporadic CJD***				
MM1	43	40	3	93.0
VV2	33	33	0	**100.0**
MV2K	26	24	2	**92.3**
MM2C	9	6	3	66.7
MM2T^§^	4	3	1	**75.0**
VV1	1	1	0	—
***genetic CJD***				
E200K-129M	20	20	0	**100.0**
V210I-129M	10	10	0	**100.0**
D178N-129V	1	1	0	—
R208H-129V	1	1	0	—
E219G-129V	1	1	0	—
***Other variants***				
GSS (P102L)	3	1	2	**25.0**
GSS (D202N)	1	0	1	—
GSS (A117V)	1	1	0	—
GSS (ins-8 repeats)	1	0	1	—
FFI	2	0	2	0
VPSPr	3	3	0	100.0
vCJD	4	1	3	**25,0**
iCJD	2	2	0	**100.0**
***probable sporadic CJD***				
MM	39	38	1	97.4
MV	24	22	2	91.7
VV	10	9	1	90.0

^§^Include one probable case (see also methods).

## Discussion

In this study we have applied IQ-CSF, the second generation RT-QuIC prion assay, to a patient cohort including the largest population analysed to date affected by the less common disease subtypes. Furthermore, we provide a direct comparison between the first and second generation prion RT-QuIC, taking advantage of a very large group of samples from definite CJD cases previously tested under PQ-CSF conditions^27^. The results confirm that, while PQ-CSF and IQ-CSF show a comparable performance in terms of specificity, IQ-CSF is associated with a remarkable increase in sensitivity, allowing the detection of 81% of CJD samples tested negative by PQ-CSF. Since in those CSF samples which tested negative by PQ-CSF, the mean seeding activity as detected by IQ-CSF was lower than in samples which tested positive under both conditions, the collected data clearly indicate that this novel assay is associated with a lower limit of detection for seeding prions. Moreover, they confirm that the added value of IQ-CSF goes beyond the crude numbers reflecting the increased sensitivity. Indeed, besides the faster response allowing a ~40-h reduction in average detection time for positive samples in comparison to PQ-CSF, IQ-CSF is also associated with a stronger fluorescence readings and a significant reduction of unclear or inconclusive results (*e.g*. when the well repeats repeatedly give an equivocal response).

A critical issue concerning the evaluation of any novel diagnostic assay for prion disease is related to the extent of clinical heterogeneity shown by these patients. This is a major point of difficulty in the clinical diagnosis and differentiation of prion diseases against other neurological disorders, not only because of the broad spectrum of clinical situations where the suspicion of prion disease can be raised, but also because the accuracy of diagnostic assays often varies significantly according to the disease subtype. This has been shown, not only for surrogate biomarkers such as proteins 14-3-3 and t-tau, but also for PQ-CSF^27^. Thus, a critical question is how well IQ-CSF detects and differentiates the less common and often atypical prion disease variants. Our results show that the high sensitivity of IQ-CSF extends to the majority of human prion strains. Most significantly, we demonstrated in the largest series of definite cases analyzed to date (n = 59), a 98% diagnostic sensitivity for the two subtypes associated with the V2 prion strain (VV2 and MV2K subtypes comprising about 30% of all sCJD cases). This is significant in that it will not only raise the overall accuracy of routine diagnosis but also improve the detection and diagnosis of these atypical subtypes. Due to the relatively frequent atypical clinical onset and/or the relatively slow disease progression (MV2K subtype), in such cases the clinical diagnosis of probable sCJD, when reached, is often delayed. In a recent study^32^, we found that about 20% of VV2 cases manifest symptoms and signs limited to the cerebellar/visual domain at time of first hospitalization and therefore do not fulfill current clinical criteria for possible sCJD, which are required to implement a diagnosis of probable sCJD based on surrogate CSF biomarkers and/or MRI findings^18^. Similarly, the diagnosis of probable sCJD in subjects with the MV2K subtype is reached, on average, only seven months after clinical onset (P.Parchi and coll., unpublished observation).

As far as the other sCJD subtypes are concerned, which are all very rare, our results show a lower sensitivity of the IQ-CSF for the MM2C and, possibly, MM2T. Most significantly, for MM2C our results are in line with those of Foutz *et al*.^29^, namely showing lower values in both assay sensitivity and seeding effect in MM2C in comparison to MM1. By combining our 9 cases with the 9 analyzed by Foutz *et al*., the overall calculated sensitivity of IQ-CSF for sCJD MM2C is 68%. Since M2C prions have a significantly reduced transmission efficiency relative to M1 prions in the most compatible host genotype^33^, these results may indicate that the response detected by IQ-CSF reflects such a property of prions. Indeed, previous studies, combining brain sample dilutions and/or the use of different substrates have already demonstrated the potential of RT-QuIC for prion strain discrimination^34, 35^. However, whether these preliminary data reflect differences in abnormal PrP concentrations, strain-specific PrP^Sc^ properties or both remain to be seen. Thus, additional work with each type of human prions will be required to better establish the quantitative relationships between RT-QuIC seeding activity and the levels of PrP^Sc^ in tissue samples.

Finally, although with data obtained from a limited number of samples, we demonstrated here for the first time that the IQ-CSF may detect prion seeding activity even in VPSPr, a highly distinctive prion disease phenotype, which is notoriously associated with a low sensitivity of surrogate biomarker assays^6^. Furthermore, as for sCJD MM2C, we detected a significantly lower seeding effect in VPSPr in comparison to the most common sCJD prions (e.g. M1 and V2 strains), further supporting the idea that IQ-CSF may, with the limitations outlined above, differentiate between prions with relatively high or low propagation efficiency (in their most compatible host genotype).

The present results, combined with those of other recent studies^29–31^, demonstrate that prion CSF RT-QuIC is not only fully specific, but also has even higher sensitivity than the surrogate biomarker assays making it the most powerful currently available tool for the clinical diagnosis of prion disease in humans. Given the high diagnostic performance of IQ-CSF, the role of surrogate CSF markers in the differential diagnosis of rapidly progressive neurological syndromes should be reconsidered. If a complementary role of such assays beside RT-QuIC should be envisaged, the choice should definitely go towards biomarkers such as t-tau or, perhaps, the neurofilament proteins^36^ (which are increasingly used in the differential diagnosis of neurodegenerative dementia), rather than 14-3-3, a biomarker whose application has been limited to the differential diagnosis of CJD. Indeed, in case where the RT-QuIC assay is negative, the result of the t-tau assay may be helpful in re-directing the diagnostic work-up.

In conclusion, the results of the present study strongly support the rapid implementation of IQ-CSF testing in clinical practice. By rapidly confirming or excluding the CJD diagnoses with high accuracy the assay is expected to improve the outcome for patients as well as their enrollment in future therapeutic trials.

## Methods

### CSF samples and case classification

We retrospectively analyzed CSF samples from 339 patients, including 282 samples sent for diagnostic purpose to the Laboratory of Neuropathology (NP-Lab), Institute of Neurological Sciences of Bologna (ISNB), 50 samples collected at the IRCCS Foundation Carlo Besta Neurological Institute (INCB), and 7 samples collected at the National CJD Research and Surveillance Unit of the UK. The study was conducted according to the revised Declaration of Helsinki and Good Clinical Practice guidelines and regulations. Informed consent was given by study participants or their next of kin. Data collection and CSF sample analysis of clinically suspected cases in Italy is an integral part of the National CJD surveillance study, which was approved by the Ethic Committee of the Istituto Superiore di Sanità (CE-ISS 09/266 on 29 may 2009).

CSF samples were collected by lumbar puncture (LP) following a standard procedure, centrifuged at 1000 × g for 10 min and stored in polypropylene tubes at −80 °C until analysis.

Molecular analysis of the *PRNP* gene was performed in all subjects as previously described^37^. All definitive cases were classified based on histopathological features, *PRNP* genotype and PrP^Sc^ type, as described^9, 27, 38^. The 125 tested definite (sporadic or acquired) CJD included 43 MM(V)1 or MM(V)1+2 C (abbreviated in the manuscript as MM1), 33 VV2, 26 MV2K or MV2K+2 C (abbreviated as MV2K), and 23 cases of rarest subtypes (4 MM2T, 9 MM2C, 3VPSPr, 1VV1, 4 vCJD and 2 dura-mater associated iCJD MM1). The 73 probable sCJD cases were classified based on codon 129 genotype and included 39 MM, 24 MV and 10 VV subjects. A single case was classified as probable MM2T based on typical clinical, polysomnographic and PET findings^39^ and added to the MM2T group (n = 4) (Tables 1 and 3). The 41 genetic cases included the following *PRNP* haplotypes/mutations: E200K-129M (n = 20), V210I-129M (n = 10), D178N-129M, D178N-129V, R208H-129M, P102L-129M, E219G-129V, D202N, A117V, ins-8 repeats (all n = 1). Finally, the non-CJD group included 100 cases with clinical features compatible with CJD, in which post-mortem findings, including histopathological and PrP western blot analysis, or the results of laboratory investigation at follow-up excluded the diagnosis of prion disease (Table 2). Specifically, definitive clinical diagnosis included a) neurodegenerative diseases linked to a proven pathogenic mutation, b) autoimmune encephalitis with anti-(onco)neuronal antibodies (paraneoplastic encephalitis) or membrane-associated antineuronal antibodies (non-paraneoplastic autoimmune encephalitis) in serum and/or CSF, and infectious encephalitis confirmed by laboratory microbiological findings and c) metabolic/toxic encephalopathies confirmed by clinical (follow-up), neuroradiological and laboratory findings.

### PrP^Sc^ detection by RT-QuIC

CSF samples were analyzed by the RT-QuIC assay with two different protocols. The PQ-CSF protocol was previously described^27^. For IQ-CSF protocol, 15 µl of each CSF sample were added in the dark to 85 µl of reaction mix in black, clear-bottom, 96-well microplates. Samples were tested in quadruplicate together with positive (definite CJD) and negative (non-CJD) controls. The RT-QuIC reaction mix contained 10 mM phosphate buffer at pH 7.4, 300 mM NaCl, 1 mM ethylenediaminetetraacetic acid tetrasodium salt dihydrate (EDTA) at pH 8.0, 10 μM thioflavin-T (ThT), 0.002% of Sodium dodecyl sulfate (SDS) and 0.1 mg/ml of Syrian hamster recombinant truncated form of prion protein (Ha rPrP 90–231)^28^. All the reaction solutions were freshly prepared and filtered before use with 0.22 µm sterile filters. After sealing, the plate was incubated in a FLUOstar OMEGA reader (BMG Labtech, Germany) at 55 °C, over a period of 60 hours with intermittent cycles of shaking (60 s, 700 rpm, double-orbital) and rest (60 s). The fluorescence intensity of ThT-PrP^Sc^ aggregates, expressed as relative fluorescence units (RFU), was taken every 45 minutes using 450 ± 10 nm (excitation) and 480 ± 10 nm (emission) wave-lengths, with a bottom read and a gain of 1000. A CSF sample was considered prion positive if at least two out four sample replicates gave a fluorescence signal higher than the threshold cut-off value. This threshold represents the mean RFU values of negative samples plus at least 10 standard deviations. Samples were considered negative if none of the replicates surpassed the chosen cut-off. In two cases, only one of four replicate went over the threshold, thus the test was considered ambiguous/unclear and repeated.

### 14-3-3 protein detection and t-tau quantification in CSF

These assays were performed as described^27^. Briefly, protein 14-3-3 was evaluated semi-quantitatively by comparing the Western Blot signals of the tested sample in comparison to those of control samples (with a weak or a strong 14-3-3 signal, respectively). T-tau protein was quantitatively analyzed using commercially available kits based on a sandwich ELISA method, according to the manufacturer’s instructions (INNOTEST, Innogenetics, Gent, Belgium). Based on previous analyses^27^, the cut-off value chosen for t-tau was 1250 pg/ml.

### Statistical analyses

RT-QuIC relative fluorescence responses were analysed and plotted using the Sigma Plot software (Systat Software Inc, Chicago, IL, USA). Depending on the data distribution, the Mann-Whitney test were used to test differences between two groups, while the one-way ANOVA (followed by Tukey’s or Bonferroni’s post hoc test) were applied for multiple group comparisons. P values < 0.05 is considered statistically significant. Unless otherwise stated, data are expressed as mean with standard error of the mean (SEM) and number of sample tested (n).

### Data availability

The datasets generated during and/or analyzed during the current study are available from the corresponding author on reasonable request.

## Electronic supplementary material

Supplementary information
