# Supplementary material for: High diagnostic value of second generation CSF RT-QuIC across the wide spectrum of CJD prions
Source: Sci Rep. 2017 Sep 6;7:10655. doi: 10.1038/s41598-017-10922-w (PMC5587608; doi:10.1038/s41598-017-10922-w)
Supplement: Supplementary file 1 — Supplementary information [file 41598_2017_10922_MOESM1_ESM.docx]

**Supplementary information.**

Manuscript title: **High diagnostic value of second generation CSF RT-QuIC across the wide spectrum of CJD prions**

Authors: Alessia Franceschini, Simone Baiardi, Andrew G. Hughson, Neil McKenzie, Fabio Moda, Marcello Rossi, Sabina Capellari, Alison Green, Giorgio Giaccone, Byron Caughey, and Piero Parchi.

**Suppl. Table 1.** Direct comparison between PQ-CSF and IQ-CSF RT-QuIC assays.

| **PQ-CSF** | **IQ-CSF** | | | |
| --- | --- | --- | --- | --- |
| **N of positive wells (samples, n)** | **N of positive wells (samples, n)**  **%** | | | |
| 0 (58^a^) | 0 (11) | 2 (6) | 3 (6) | 4 (35) |
|  | **19.0%** | **10.3%** | **10.3%** | **60.3%** |
|  |  |  |  |  |
| 2 (35^b^) | 0 (1) | 2 (1) | 3 (1) | 4 (32) |
|  | **3.0%** | **3.0%** | **3.0%** | **91.0%** |
|  |  |  |  |  |
| 3 (35^c^) | 0 (0) | 2 (0) | 3 (2) | 4 (33) |
|  | **0.0%** | **0.0%** | **6.0%** | **94.0%** |
|  |  |  |  |  |
| 4 (35^d^) | 0 (0) | 2 (0) | 3 (2) | 4 (33) |
|  | **0.0%** | **0.0%** | **6.0%** | **94.0%** |

^a^28 definite sCJD, 6 gCJD and 24 probable sCJD; ^b^24 definite sCJD, 7 genetic and 4 probable

sCJD; ^c^26 definite sCJD, 4 gCJD and 5 probable sCJD; ^d^30 definite sCJD and 5 gCJD

**
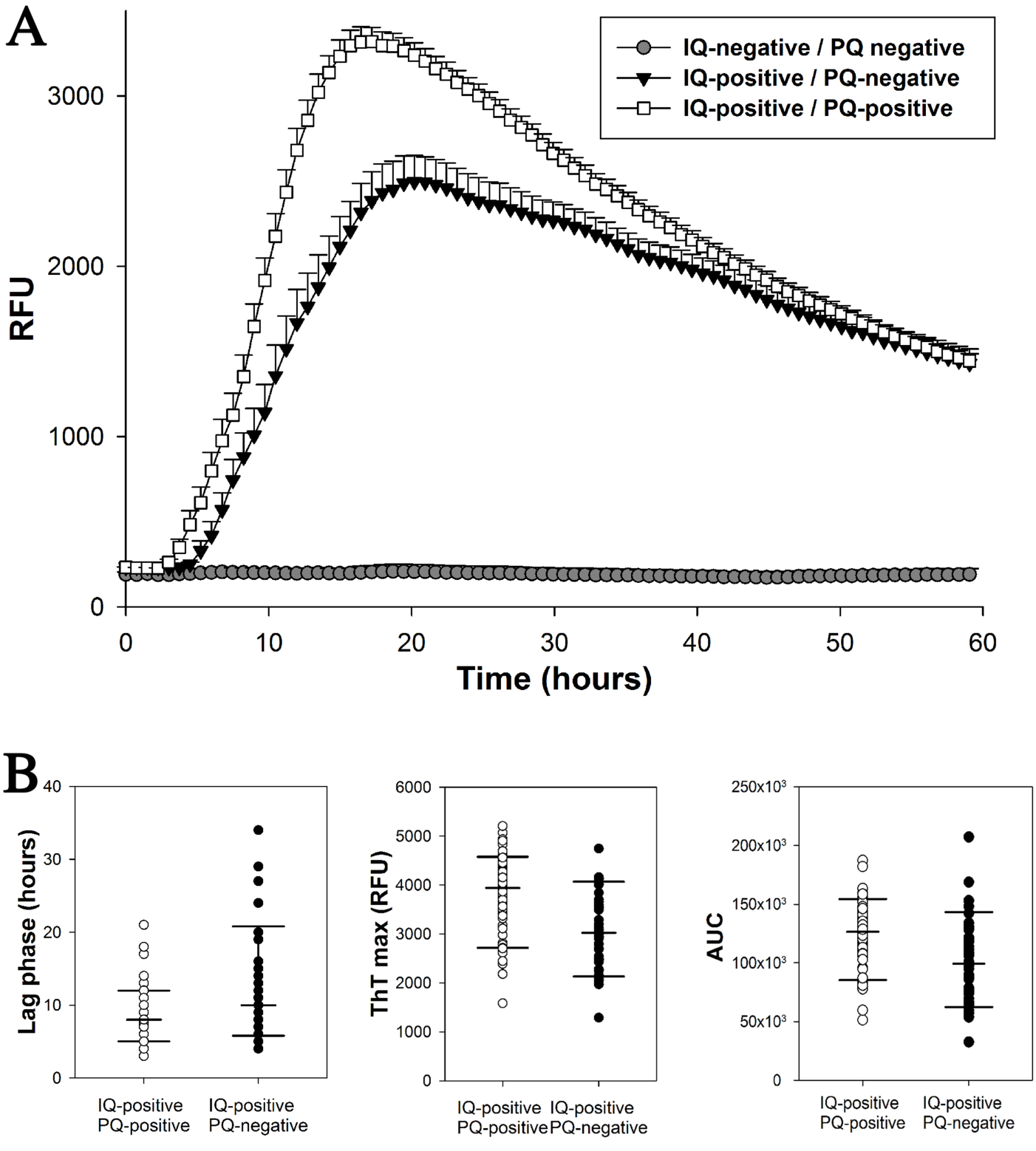
**

**Suppl. Figure 1. Comparison of IQ-CSF kinetics and time to threshold between PQ-positive and PQ-negative CJD samples.**  (**A**) Traces represent the mean ± SEM of ThT fluorescence for CJD CSFs grouped as follows: (i) PQ- and IQ-negative, black, n=11, (ii) PQ-negative and IQ-positive, gray, n=47; and (iii) PQ- and IQ positive, white, n=104. (**B**) Comparison of lag phases, ThT max and AUC under IQ conditions between PQ-positive and PQ-negative CJD samples (p< 0.05, p<0.001, and p<0.001, respectively).

**Suppl. Table 2.** IQ-CSF lag phases, ThT max and AUC for groups not included in Figure 2.

|  | **n** | **Lag phase (Hours)** | **ThT max (RFU)** | **AUC** |
| --- | --- | --- | --- | --- |
| **gCJD E219G-129V** | 1 | 16 | 2259 | 75300 |
| **gCJD R208H-129V** | 1 | 10 | 2249 | 86160 |
| **gCJD D178N-129V** | 1 | 21 | 2545 | 78800 |
| **GSS P102L** | 1 | 12 | 2250 | 74030 |
| **GSS A117V** | 1 | 12 | 1953 | 62800 |
| **sCJD VV1** | 1 | 7 | 3500 | 137000 |
| **iCJD** | 2 | 15±3 | 3500±200 | 115700±400 |
| **vCJD** | 1 | 14 | 3250 | 90260 |

|  | **IQ-CSF** | | | **t-tau** | | | **14-3-3** | | |
| --- | --- | --- | --- | --- | --- | --- | --- | --- | --- |
|  | **Positive/**  **Total** | **Sensitivity (%)** | **Specificity (%)** | **Positive/**  **Total** | **Sensitivity (%)** | **Specificity (%)** | **Positive/**  **Total** | **Sensitivity (%)** | **Specificity (%)** |
| **sCJD MM(V)1** | 39/41 | **95.1** |  | 39/41 | **95.1** |  | 39/41 | **95.1** |  |
| **sCJD VV2** | 13/13 | **100** |  | 13/13 | **100** |  | 13/13 | **100** |  |
| **sCJD MV2K** | 9/9 | **100** |  | 6/9 | **66.6** |  | 4/9 | **44.4** |  |
| **probable sCJD MM** | 36/37 | **97.3** |  | 36/37 | **97.3** |  | 35/37 | **94.6** |  |
| **probable sCJD MV** | 16/17 | **94.1** |  | 16/17 | **94.1** |  | 12/17 | **70.6** |  |
| **probable sCJD VV** | 7/7 | **100** |  | 7/7 | **100** |  | 7/7 | **100** |  |
| **gCJD** | 21/21 | **100** |  | 19/21 | **90.5** |  | 16/21 | **76.2** |  |
| **Definite non-CJD** | 0/42 |  | **100** | 16/42 |  | **62.0** | 18/42 |  | **57.2** |

**Suppl. Table 3.** Comparison of diagnostic performance of IQ-CSF RT-QuIC, t-tau and 14-3-3 assays in a consecutive cohort of definite and probable CJD and definite non-CJD cases examined at NP Lab (time period 2011-2017).
